# Dysmagnesemia in the ICU: A Comparative Analysis of Ionized and Total Magnesium Levels and Their Clinical Associations

**DOI:** 10.3390/metabo15080498

**Published:** 2025-07-24

**Authors:** Jawahar H. Al Noumani, Juhaina Salim Al-Maqbali, Mohammed Al Maktoumi, Qasim Sultan AL-Maamari, Abdul Hakeem Al-Hashim, Mujahid Al-Busaidi, Henrik Falhammar, Abdullah M. Al Alawi

**Affiliations:** 1 Department of Medicine, Sultan Qaboos University Hospital, University Medical City, P.O. Box 141, Muscat 123, Omanhashim@squ.edu.om (A.H.A.-H.); mujahidalbusaidi@gmail.com (M.A.-B.); 2 College of Medicine and Health Science, Sultan Qaboos University, P.O. Box 141, Muscat 123, Oman; jsmm14@gmail.com; 3 Department of Pharmacy, Sultan Qaboos University Hospital, University Medical City, P.O. Box 141, Muscat 123, Oman; 4 Internal Medicine Residency Program, Oman Medical Specialty Board, P.O. Box 141, Muscat 123, Oman; r2179@resident.omsb.org; 5 Department of Nursing Department, Sultan Qaboos University Hospital, University Medical City, P.O. Box 141, Muscat 123, Oman; qasim22@squ.edu.om; 6 Department of Endocrinology, Karolinska University Hospital, 17176 Stockholm, Sweden; henrik.falhammar@ki.se; 7 Department of Molecular Medicine and Surgery, Karolinska Institute, 17176 Stockholm, Sweden

**Keywords:** critical care, dysmagnesemia, ionized magnesium, magnesium replacement, total magnesium, patient outcomes

## Abstract

**Background:** Magnesium (Mg) is an essential mineral that plays a vital role in various physiological processes, including enzyme regulation, neuromuscular function, and cardiovascular health. Dysmagnesemia has been associated with arrhythmias, neuromuscular dysfunction, and poor outcomes in intensive care unit (ICU) settings, representing diagnostic and therapeutic challenges. However, the relationship between dysmagnesemia and health outcomes in the ICU remains inadequately defined. **Aim/Objective:** This study aimed to assess the prevalence of dysmagnesemia and evaluate the correlation between total (tMg) and ionized magnesium (iMg) levels in a cohort of ICU and high dependency unit (HDU) patients. It also sought to evaluate patient characteristics and relevant health outcomes by comparing both concentrations of iMg and tMg. **Methods:** This prospective study was conducted among adult patients admitted to the ICU and the high dependency unit (HDU). **Results:** Among the 134 included patients, the median age was 63.5 years (IQR: 52.0–77.0). The majority, 91.0%, required mechanical ventilation. Additionally, 50.0% were diagnosed with diabetes, 28.4% had chronic kidney disease, and proton pump inhibitors (PPIs) were administered to 67.2% of the patients. The prevalence of hypomagnesemia, as measured by iMg, was 6.7%, while hypermagnesemia was at 39.6%. When measured by tMg, hypomagnesemia and hypermagnesemia were observed at rates of 14.9% and 22.4%, respectively. The iMg measurements showed an association between the incidence of atrial fibrillation and hypomagnesemia (*p* = 0.015), whereas tMg measurements linked hypomagnesemia with longer hospital stays. Notably, only a few patients identified with iMg-measured hypomagnesemia received magnesium replacement during their ICU stay. **Conclusions:** Dysmagnesemia is prevalent among critically ill patients, with discordance between iMg and tMg measurements. iMg appears more sensitive in detecting arrhythmia risk, while tMg correlates with length of stay. These findings support the need for larger studies and suggest considering iMg in magnesium monitoring and replacement strategies.

## 1. Introduction

Magnesium (Mg) is a crucial mineral involved in numerous physiological processes, including enzyme regulation, neuromuscular function, and cardiovascular health. In the context of critical illness, Mg disorders, particularly hypomagnesemia, are common and have significant implications for patient outcomes in the intensive care unit (ICU) [1]. Hypomagnesemia is prevalent among patients in the ICU, with reported rates ranging from 20% to 65% [1,2,3,4,5,6,7]. Several factors contribute to hypomagnesemia in critically ill patients, including reduced intestinal absorption, nasogastric suction, and insufficient Mg content in total parenteral nutrition [8]. Additional contributors are disturbances in acid–base balance and the use of certain medications known to potentially cause hypomagnesemia, such as proton pump inhibitors (PPIs), diuretics, and aminoglycosides [2,3]. In the setting of the ICU, hypomagnesemia is associated with many poor health outcomes including increased mortality, prolonged length of ICU stay and the need for mechanical ventilation [3]. Hypomagnesemia is commonly associated with other conditions such as sepsis and diabetes mellitus, which are prevalent in critically ill patients [4].

Hypermagnesemia was found in 9% of patients in the ICU according to a study measuring serum Mg concentrations in consecutive ICU admissions (n = 102) [6]. Another study reported that 14% of patients in the ICU had hypermagnesemia measured by ionized Mg (iMg) at admission (n = 446) [9]. Recent studies suggest that hypermagnesemia is associated with many adverse health outcomes among patients hospitalized in medical wards. [2,10,11,12,13,14,15] However, hypermagnesemia is poorly studied among critically ill patients [10,16].

Most previous studies have measured the total serum Mg concentration (tMg) [1,2,3,4]. However, 25% of extracellular Mg is bound to albumin, 8% is complexed with anions, and between 65 and 70% exists in a free and bioactive ionized form (iMg) [11,17,18]. Therefore, tMg may not be a reliable indicator of Mg status, especially among critically ill patients, whose conditions change rapidly. iMg is considered a more accurate indicator of physiological magnesium status, especially in critically ill patients where protein levels and acid–base disturbances can alter magnesium binding. However, due to the limited availability of iMg measurements in many ICUs, tMg is still more commonly used in practice [19].

Given the clinical relevance of dysmagnesemia and the diagnostic ambiguity between iMg and tMg measurements, this study aimed to (1) determine the prevalence of dysmagnesemia in ICU patients, and (2) explore the correlation between iMg and tMg, which may guide future diagnostic approaches. Furthermore, the study aimed to evaluate patient characteristics and relevant health outcomes such as duration of stay in high acuity areas, in-hospital mortality, time spent on ventilator, cardiovascular events, re-admission within 90 days, and mortality within 90 days.

## 2. Materials and Methods

### 2.1. Study Design, Setting, and Population

This is a prospective cohort study that took place at Sultan Qaboos University Hospital (SQUH), a tertiary hospital with specialized health care services [20]. The study included adult patients admitted to the intensive care unit (ICU) and the high dependency unit (HDU) between 15 March 2023 and 31 December 2023. For patients with multiple admissions within 90 days, only the initial admission was included in the study and subsequent admissions were excluded. Patients without complete follow-up data were also excluded from the study.

### 2.2. Data Collection

We trained research assistants to handle consent, blood collection, and data gathering. For each patient, a venous blood sample of 1–2 mL was drawn using a tourniquet and heparinized syringes loaded with 60 U heparin, obtained from Radiometer^®^, located in Bronshoj, Denmark. For the assessment of ionized constituents, the samples were analyzed with an electrolyte analyzer, Stat Profile Prime Plus^®^, supplied by Nova Biomedicals in Waltman, MA, USA. This device utilizes the direct ISE method, which measures the concentration of iMg using a neutral carrier-based membrane with an ionophore that selectively accommodates the size of the Mg ion. To maintain sample stability, all samples underwent analysis either immediately or within an hour after being collected [19].

The tMg concentration was assessed using a colorimetric end-point reaction between Mg and xylidyl blue in an alkaline solution. This examination was carried out with the help of a Roche Cobas modular analyzer in SQUH’s Biochemistry Department [14].

Measurements of tMg, iMg, and other electrolytes were taken upon admission to the ICU and HDU. Additional data collected included demographic details, comorbidities, risk factors, medications, laboratory results, treatments for dysmagnesemia, and outcomes. Follow-up information was obtained by accessing electronic health information systems, or via phone calls to either the patient or their next of kin.

In this ICU setting, magnesium replacement decisions were generally based on tMg levels, as tMg is routinely reported and more accessible than iMg. There was no standardized institutional protocol for magnesium supplementation. Treatment decisions were made at the discretion of the attending clinician, considering both the magnesium level and the broader clinical context, including symptoms, comorbidities, clinical status of the patient, and other electrolyte abnormalities.

### 2.3. Definitions

Patients were classified into three groups based on their serum magnesium levels at ICU admission using institutional laboratory reference thresholds. For tMg, hypomagnesemia was defined as ≤0.69 mmol/L, normomagnesemia as 0.70–1.00 mmol/L, and hypermagnesemia as ≥1.01 mmol/L L [14]. The local Omani population’s established reference range for iMg concentrations was used to identify iMg level, hypomagnesemia was defined as ≤0.46 mmol/L, normomagnesemia 0.47–0.68 mmol/L, and hypermagnesemia as ≥0.69 mmol/L [19]. Data for both tMg and iMg were analyzed in subgroups to assess concordance and discordance between the two parameters. This dual approach allowed for a comparison of the prognostic value of each measure and an exploration of potential differences in clinical implications.

### 2.4. Sample Size

In ICU settings, the previously reported prevalence of hypomagnesemia ranged from 20% to 65% [1,2,3,4]. The prevalence of hypermagnesemia ranged from 9% to 14% [6,9]. We estimated that we would need a minimum sample size of 125 patients to estimate the prevalence of hypermagnesemia in our setting and 240 patients to estimate the prevalence of hypomagnesemia to achieve a 95% confidence interval with 80% statistical power (Alpha 0.05, Beta 0.2).

### 2.5. Ethical Approval

This study was ethically approved by the Medical Research Ethics Committee (MREC), College of Medicine and Health Sciences, Sultan Qaboos University (*SQU-EC/031/2023*, MREC #2960) on 28 February 2023. Written consent was secured either directly from the patients or, when the patients’ capacity was impaired, from their next of kin.

### 2.6. Statistical Analysis

Categorical variables are presented as frequencies and percentages. Continuous variables are expressed as mean ± SD for normally distributed variables and median (interquartile ranges; IQRs) for non-normally distributed variables. The Kruskal–Wallis test determined the relationship between variables and different iMg or tMg concentration groups. The chi-square test was performed to examine the relationships between categorical variables and different Mg concentration groups, while Fisher’s exact test was applied if the cells had an anticipated frequency of less than five. Multivariable logistic regression models were built using a backward stepwise approach to identify independent predictors of clinical outcomes (e.g., arrhythmia, 90-day mortality, ICU readmission) based on magnesium categories and patient characteristics. Variables with *p* < 0.10 in univariate analyses were considered for inclusion. Lastly, time-to-event survival analysis for 90-day re-admission and 90-day all-cause mortality was performed using the Kaplan–Meier method, and log-rank tests for comparisons among patients in the three different groups of iMg concentration to the identify hazard ratio (HR). The two-tailed level of significance was set at *p* < 0.05 level. Statistical analyses were conducted using STATA version 17.0 (STATA Corporation, College Station, TX, USA).

## 3. Results

A total of 134 patients were enrolled. A post hoc power analysis was conducted using binomial tests based on the observed and expected prevalence rates and a sample size of 134. The study had an estimated power of 81.2% to detect a difference in hypomagnesemia prevalence from the expected 14.4%, and 98.0% power for hypermagnesemia compared to the expected 23%.

Out of 134 patients, 53 (39.6%) were female. The median age was 63.5 (IQR: 52.0–77.0) years, and out of all the patients, 122 (91.0%) were receiving mechanical ventilation, either invasive or non-invasive, and 87 (64.9%) were receiving vasopressors or inotropes. Among the included patients, diabetes mellitus (DM) was prevalent (n = 67, 50.0%), followed by cases of chronic kidney disease (CKD) (n = 38, 28.4%). Proton pump inhibitors (PPIs) were commonly prescribed (n = 90, 67.2%), followed by loop diuretics, and furosemide (n = 55, 41.0%).

The prevalence of hypomagnesemia measured by iMg concentration was 6.7% (95% CI; 3.3–12.7), and the prevalence of hypermagnesemia was 39.6% (95% CI; 31.3–48.4). While the prevalence of hypomagnesemia measured by tMg concentration was 14.9% (95% CI; 9.6–22.4), and the prevalence of hypermagnesemia was 22.4% (95% CI; 15.8–30.6).

Pearson’s correlation coefficient between iMg and tMg concentrations demonstrated a weak positive linear relationship between these two variables (r = 0.286; *p* < 0.01).

The prevalence of DM was higher in the hypomagnesemia and normomagnesemia groups measured by tMg concentration compared to the hypermagnesemia group (50.0% vs. 60.7% vs. 20.0%; *p* < 0.01) but there were no differences between the groups when measured by iMg. Similarly, the incidence of diabetic ketoacidosis was more common in the hypomagnesemia group measured by iMg concentration compared to normomagnesemia and hypomagnesemia groups (22.2% vs. 4.2% vs. 1.9%; *p* = 0.01), but there were no differences between the groups by tMg. Patients who were kept nil by mouth were more likely to be in the hypomagnesemia and normomagnesemia groups measured by iMg concentration compared to the hypermagnesemia group (77.8% vs. 77.8% vs. 54.7%; *p* = 0.019). Conversely, diabetic ketoacidosis was more common in the hypermagnesemia group measured by iMg concentration compared to normomagnesemia and hypomagnesemia groups (33.8% vs. 20.8% vs. 23.5%; *p* = 0.024), but there were no differences between the groups by tMg (Table 1 and Table 2).

Serum sodium concentrations were higher among patients with hypermagnesemia in the group measured by iMg compared to the other groups (144.4 vs. 139.3 vs. 135.9; mmol/L, *p* < 0.01), while it was not the case with the groups measured by tMg concentrations. However, serum chloride concentration showed a similar pattern, where its concentration was lower in hypomagnesemia in both groups measured by iMg and tMg concertation, respectively (Table 1 and Table 2).

During ICU/HDU admission, 39 patients (29.1%) received Mg replacement. Among the hypomagnesemia group measured by iMg concentration, only 3 patients (33.3%) received Mg replacement, which was much lower than the patients who were treated in the hypomagnesemia group measured by tMg concentration (n = 11, 55.0%) (Table 1 and Table 2).

**Figure 1:**
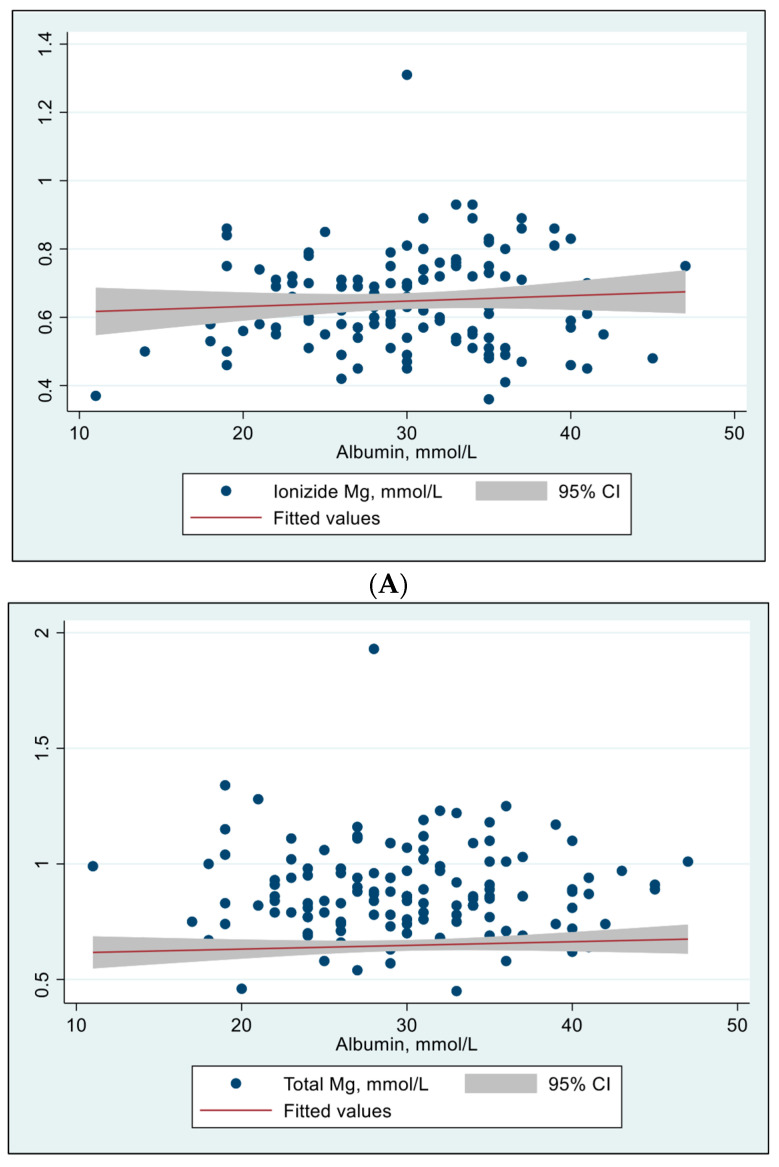
Effect of albumin concentrations on ionized Mg and total Mg concentrations (N = 134). (**A**) Effect of albumin concentration on ionized Mg concentration. Spearman’s rho = 0.061, *p* = 0.483. Person’s (*r*) = 0.079, *p* = 0.361. (**B**) Effect of albumin concentration on total Mg concentration. Spearman’s rho = –0.019, *p* = 0.828. Person’s (*r*) = –0.029, *p* = 0.733.

The relationship between albumin concentrations and iMg and tMg concentrations was evaluated using Spearman’s rho test and were all insignificant (Figure 1).

As shown in Table 3, in terms of clinical outcomes, when the Mg was measured by iMg concentrations, the incidence of atrial fibrillation was associated with hypomagnesemia (44.4%, *p* = 0.015) when compared to higher concentrations.

The incidence of all types of arrhythmias was higher in the hypomagnesemia group compared to the normomagnesemia group (55.6% vs. 33.3% vs. 11.3%; *p* < 0.01). However, this association was not observed when magnesium levels were measured using tMg concentrations (Table 4). Multivariate analysis did not identify iMg levels as an independent predictor for atrial fibrillation (AF) (Odds Ratio [OR]: 0.08; *p* = 0.74; 95% CI: [3.76–189429.1]) or for all types of arrhythmias (OR: 0.02; *p* = 0.59; 95% CI: [6.84–46572.2]) in the regression analysis.

In contrast, the hypomagnesemia group measured by tMg concentrations showed a longer length of stay among other groups (23.5 vs. 12.0 vs. 13.0 days; *p* < 0.01). Moreover, Table 5 displays the calculated fraction of iMg to tMg concentrations. The results showed a significant association with length of stay when the fraction exceeded 60%.

**Figure 2:**
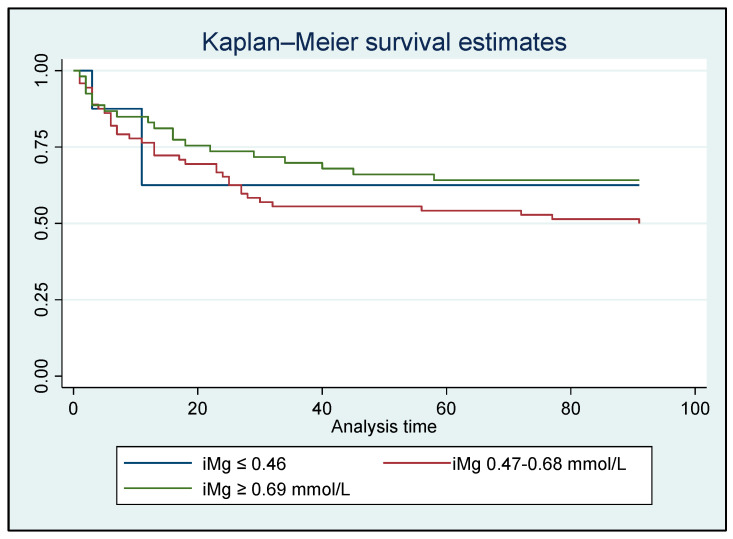
Kaplan–Meier analysis for in-hospital/90-day all-cause mortality based on ionized Mg concentrations for all patients who survived (N = 134); HR: 0.78, *p* = 0.281 [95% CI: 0.51–1.21].

**Figure 3:**
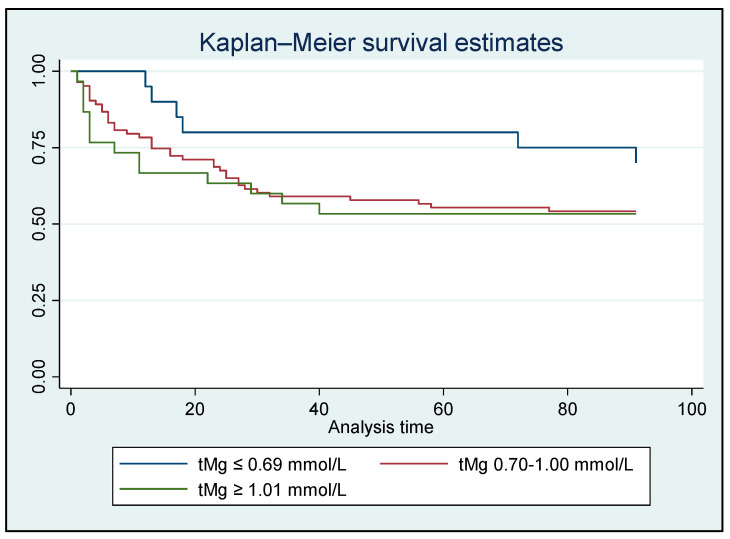
Kaplan–Meier analysis for in-hospital/90-day all-cause mortality based on total Mg concentrations for all patients who survived (N = 134); HR: 1.322, *p* = 0.191 [95% CI: 0.87–2.01].

A time-to-event survival analysis for in-hospital/90-day all-cause mortality was conducted using both iMg and tMg concentrations. However, no significant difference was found between the Mg groups (Figure 2 and Figure 3).

## 4. Discussion

The study is the first study that provides data using both iMg and tMg to evaluate the prevalence of dysmagnesemia and related health consequences among patients admitted to the ICU/HDU. Note that the study involved mainly elderly patients who were critically ill, with most of them being mechanically ventilated, receiving vasopressors, and most were administered PPIs. The study showed a weak positive linear relationship between iMg and tMg concentrations. Among the cohort, the prevalence of hypomagnesemia by iMg concentration was lower than when it was measured by tMg concentration. While the prevalence of hypermagnesemia by iMg concentration was higher than when it was measured by tMg concentration. The occurrence of atrial fibrillation and various types of arrhythmias were associated with the hypomagnesemia group measured by iMg concentration. On the other hand, the group with hypomagnesemia, as indicated by tMg concentrations, had a longer duration of hospitalization.

Pearson’s correlation coefficient analysis showed a weak positive linear relationship between iMg and tMg concentrations. Very few studies have explored the correlation between iMg and tMg concentrations, yielding conflicting results that vary depending on the specific medical conditions and the measurement methods used [21]. iMg is the physiologically active form of magnesium, whereas tMg includes iMg, protein-bound Mg (pbMg), and complexed Mg (cMg). Several factors such as tissue damage, dietary intake, and severe illness might cause a poor correlation between iMg and tMg [22,23].

The prevalence of hypomagnesemia measured by iMg concentration was 6.72%, which is comparable to the previously reported prevalence of hypomagnesemia measured by iMg concentration among patients in the ICU (9.7%–14.4%) [24,25]. As anticipated, most patients were administered PPIs for stress ulcer prophylaxis. PPIs are known to induce hypomagnesemia by impeding gastrointestinal Mg absorption [26]. The prevalence of hypermagnesemia measured by iMg was 39.55%, which is higher than the previously reported prevalences among patients in the ICU (14–23%) [9,24]. The variations in these prevalence rates might be explained by the different reference ranges used to define normal iMg. Our reference range for iMg, identified for the local population, was between 0.47 and 0.68 mmol/L [19]. The prevalence of hypomagnesemia measured by tMg concentration was 14.93%. Previous reports showed variable prevalences of hypomagnesemia measured by tMg in various ICU settings, which ranged between 11% and 62% [27]. The prevalence of hypermagnesemia measured by tMg was 22.39%. Unlike hypomagnesemia, hypermagnesemia has been poorly studied in previous reports [6]. The variability in the prevalence of dysmagnesemia among patients in ICUs across different studies can be attributed to differences in patient populations, underlying conditions, measurement methods, medication use, and the severity of illness [28,29]. Standardizing the definitions and measurement techniques for hypomagnesemia, along with considering patient-specific factors, can help in better understanding and managing this common electrolyte disturbance in critically ill patients.

The prevalence of DM was higher in the hypomagnesemia and normomagnesemia groups measured by tMg concentration compared to the hypermagnesemia group, but there were no differences between the groups when measured by iMg. Hypomagnesemia at the time of ICU admission is associated with high mortality in critically ill patients with type 2 diabetes [30].

Diabetic ketoacidosis was more common in the hypomagnesemia group when measured by iMg concentration, compared to the normomagnesemia and hypermagnesemia groups. However, there were no differences between the groups when assessed by tMg. This may be because conventional treatment for diabetic ketoacidosis results in a progressive decrease in iMg and calcium concentrations, indicating a state of depletion during recovery [31].

Our findings showed that a limited number of patients with iMg-defined hypomagnesemia received magnesium replacement. This likely reflects current clinical practice where tMg remains the primary parameter used to guide treatment. In the absence of formal institutional guidelines, supplementation is typically determined by the treating physician’s clinical judgment. This practice variation may contribute to discrepancies in treatment across iMg and tMg categories. However, due to the observational nature of our study and small sample size, we did not examine the impact of magnesium replacement on clinical outcomes [32].

The study explored the relationship between albumin concentrations and both iMg and tMg concentrations using Spearman’s rho test. Interestingly, it did not demonstrate a significant correlation between serum albumin and either iMg or tMg concentrations. This finding contrasts with prior research indicating that approximately 20–30% of circulating magnesium is bound to albumin [33]. One potential explanation is that critical illness alters the binding affinity of albumin for magnesium, possibly due to conformational changes in albumin structure related to systemic inflammation, acidosis, or oxidative stress [34]. Furthermore, other binding proteins such as globulins may contribute to magnesium transport in critical illness, but these were not measured in our cohort [35,36].

Using iMg concentrations for measurement, the study found a higher prevalence of atrial fibrillation and various types of arrhythmias associated with hypomagnesemia compared to normal or high Mg concentrations. However, these associations ceased to be noticeable when tMg concentrations were used as the measurement method. On the other hand, the hypomagnesemia group measured by tMg concentrations exhibited a longer length of stay compared to other groups. Previous studies showed that hypomagnesemia is associated with increased mortality, prolonged ICU stays, septic shock, and a higher need for mechanical ventilation [1]. Hypomagnesemia is also linked to various arrhythmias, including torsade de pointes and atrial fibrillation. Magnesium, by stabilizing myocardial ion channels, can help prevent and treat such arrhythmias. Despite this, there are still uncertainties surrounding the optimal dosage, administration methods, and long-term effects of Mg supplementation [37,38].

The calculated fraction of iMg to tMg concentrations showed a significant association with the length of stay when the fraction exceeded 60%. This association could be explained by the reduction in tMg concentration due to the impact of severe illness on protein-bound Mg (pbMg), and complexed Mg (cMg) concentrations [25].

The lack of significant differences in in-hospital or 90-day all-cause mortality between the various Mg groups, as noted in the survival analysis, is likely attributable to the limited sample size. A systematic review and meta-analysis including 1550 critically sick patients revealed a significantly increased risk of mortality among critically ill patients with hypomagnesemia (relative risk 1.90) [39].

This study’s strengths include its prospective design, use of both iMg and tMg measurements, and thorough correlation assessment. It considered factors related to dysmagnesemia and examined health outcomes like inpatient status and 90-day mortality. Limitations include the single-center setting, which affects generalizability, and the study being underpowered to assess hypomagnesemia prevalence in the ICU, with only 134 of the ideal 240 patients included. However, post hoc sample analysis indicated that the sample size was adequate to study dysmagnesemia in the ICU and was comparable to previous samples [6,7].

Inconsistent magnesium replacement, especially in iMg-defined hypomagnesemia, introduced potential treatment bias. The observational nature of the study also precludes causal conclusions between magnesium levels and clinical outcomes.

## 5. Conclusions

Dysmagnesemia was common among critically ill patients. iMg and tMg levels showed poor correlation in the critical care setting. The association between hypomagnesemia and arrhythmia appeared more pronounced when using iMg, while hypomagnesemia measured by tMg was more closely linked to a prolonged hospital stay. Notably, most patients identified with hypomagnesemia based on iMg did not receive magnesium replacement. These findings suggest that iMg may be a more sensitive or potentially useful marker for identifying arrhythmia risk in ICU patients, although further research is needed. Larger, multicenter studies and interventional trials are warranted to validate these observations and to evaluate optimal magnesium replacement strategies based on iMg thresholds, particularly in subgroups with arrhythmias or high iMg/tMg discordance.

## Figures and Tables

**Table 1:** Patient characteristics, medical histories, laboratory profiles, and medications based on ionized Mg concentrations on day of admission to ICU/HDU.

Characteristics n (%) Unless Specified Otherwise	Total 134 (100%)	≤0.46 mmol/L 9 (6.7%)	0.47–0.68 mmol/L 72 (53.7%)	≥0.69 mmol/L 53 (39.6%)	*p*-Value
Female gender, n (%)	53 (39.6%)	4 (44.4%)	28 (38.9%)	21 (39.6%)	0.950
Age; IQR, years	63.5 (52–77)	59 (45–73)	63 (53.5–73)	67 (52–80)	0.765
Patient on MIV/NIV	122 (91.0%)	8 (88.9%)	66 (91.7%)	48 (90.6%)	0.951
Patient on inotropes/vasopressors	87 (64.9%)	6 (66.7%)	49 (68.1%)	32 (60.4%)	0.669
Medical History:					
Diabetes mellitus	67 (50.0%)	3 (33.3%)	43 (59.7%)	21 (39.6%)	0.050
Chronic kidney disease	38 (28.4%)	3 (33.3%)	22 (30.6%)	13 (24.5%)	0.718
Ischemic heart disease	31 (23.1%)	1 (11.1%)	16 (22.2%)	14 (26.4%)	0.581
Previous cardiac arrhythmia	23 (17.2%)	4 (44.4%)	12 (16.7%)	7 (13.2%)	0.070
Chronic diarrhea	1 (0.7%)	0 (0.0%)	1 (1.4%)	0 (0.0%)	0.648
Recovery post AKI	44 (32.8%)	2 (22.2%)	23 (31.9%)	19 (35.8%)	0.703
Acute pancreatitis	4 (3.0%)	0 (0.0%)	3 (4.2%)	1 (1.9%)	0.655
Alcohol use	10 (7.5%)	0 (0.0%)	8 (11.1%)	2 (3.8%)	0.206
Hypercalcemia	7 (5.2%)	1 (11.1%)	4 (5.6%)	2 (3.8%)	0.647
ESRD	7 (5.2%)	0 (0.0%)	3 (4.2%)	4 (7.6%)	0.539
Diabetic ketoacidosis	6 (4.5%)	2 (22.2%)	3 (4.2%)	1 (1.9%)	0.024
Nothing per mouth	92 (68.7%)	7 (77.8%)	56 (77.8%)	29 (54.7%)	0.019
Enteral feeding	83 (61.9%)	8 (88.9%)	41 (56.9%)	34 (64.2%)	0.162
Parenteral feeding	5 (3.7%)	0 (0.0%)	3 (4.2%)	2 (3.8%)	0.824
Medications:					
Gentamicin	1 (0.7%)	0 (0.0%)	0 (0.0%)	1 (1.9%)	0.463
Amikacin	8 (6.0%)	0 (0.0%)	7 (9.7%)	1 (1.9%)	0.139
Neomycin	1 (0.7%)	0 (0.0%)	1 (1.4%)	0 (0.0%)	0.648
Paromomycin	1 (0.7%)	0 (0.0%)	1 (1.4%)	0 (0.0%)	0.648
Esomeprazole	90 (67.2%)	6 (66.7%)	50 (69.4%)	34 (64.2%)	0.823
Furosemide	55 (41.0%)	4 (44.4%)	31 (43.1%)	20 (37.7%)	0.817
Thiazide	4 (3.0%)	0 (0.0%)	3 (4.2%)	1 (1.9%)	0.655
Amphotericin B	12 (9.0%)	0 (0.0%)	9 (12.5%)	3 (5.7%)	0.259
Tacrolimus	2 (1.5%)	0 (0.0%)	2 (2.8%)	0 (0.0%)	0.417
Electrolytes:					
Albumin, mean (SD), mmol/L	30 (6.8)	29.4 (9.9)	29.7 (6.9)	30.5 (6.1)	0.818
Corrected calcium, mean (SD), mmol/L	2.16 (0.21)	2.14 (0.22)	2.17 (0.20)	2.16 (0.24)	0.879
Sodium, mean (SD) mmol/L	141.1 (8.1)	135.9 (6.6)	139.3 (7.1)	144.4 (8.5)	<0.01
Potassium, IQR, mmol/L	4.1 (3.8–4.6)	4.6 (4.4–4.9)	4.0 (3.6–4.5)	4.1 (3.9–4.5)	0.057
Chloride, IQR, mmol/L	105 (100–109)	98 (88–106)	102.5 (99.5–108)	107 (103–115)	<0.01
Phosphate, IQR, mmol/L	1.2 (0.93–1.64)	1.33 (0.96–1.7)	1.2 (0.91–1.63)	1.2 (0.95–1.78)	0.925
Hypomagnesemia Treatment (Mg replacement):					
Mg replacement (yes/NO)	39 (29.1%)	3 (33.3%)	25 (34.7%)	11 (20.8%)	0.794
Total dose of IV Mg replacement; IQR, gram	0 (0–2)	0 (0–2)	0 (0–2)	0 (0–0)	0.342

IQR: Interquartile Range; MIV: Mechanical Invasive Ventilation; NIV: Non-Invasive Ventilation; AKI: Acute Kidney Injury; ESRD: End-Stage Renal Disease; SD: Standard Deviation.

**Table 2:** Patient characteristics, medical histories, laboratory profiles, and medications based on total Mg concentrations on day of admission to ICU/HDU.

Characteristic n (%) Unless Specified Otherwise	Total 134 (100%)	≤0.69 mmol/L 20 (14.9%)	0.70–1.00 mmol/L 84 (62.7%)	≥1.01 mmol/L 30 (22.4%)	*p*-Value
Female gender	53 (39.6%)	9 (45.0%)	32 (38.1%)	12 (40.0%)	0.850
Age; IQR, years	63.5 (52–77)	61 (47–68)	67 (53–77)	59.5 (39–75)	0.1380
Patient on MIV/NIV	122 (91.0%)	19 (95.0%)	75 (89.3%)	28 (93.3%)	0.639
Patient on inotropes/vasopressors	87 (64.9%)	11 (55.0%)	55 (65.5%)	21 (70.0%)	0.545
Medical History:					
Diabetes mellitus	67 (50.0%)	10 (50.0%)	51 (60.7%)	6 (20.0%)	<0.01
Chronic kidney disease	38 (28.4%)	4 (20.0%)	27 (32.1%)	7 (23.3%)	0.438
Ischemic heart disease	31 (23.1%)	5 (25.0%)	20 (23.8%)	6 (20.0%)	0.893
Previous cardiac arrhythmia	23 (17.2%)	1 (5.0%)	19 (22.6%)	3 (10.0%)	0.085
Chronic diarrhea	1 (0.7%)	0 (0.0%)	1 (1.2%)	0 (0.0%)	0.741
Recovery post AKI	44 (32.8%)	7 (35.0%)	24 (28.6%)	13 (43.3%)	0.327
Acute pancreatitis	4 (3.0%)	0 (0.0%)	4 (4.8%)	0 (0.0%)	0.293
Alcohol use	10 (7.5%)	4 (20.0%)	5 (6.0%)	1 (3.3%)	0.062
Hypercalcemia	7 (5.2%)	1 (5.0%)	2 (2.4%)	4 (13.3%)	0.069
ESRD	7 (5.2%)	0 (0.0%)	6 (7.1%)	1 (3.3%)	0.378
Diabetic ketoacidosis	6 (4.5%)	0 (0.0%)	4 (4.8%)	2 (6.7%)	0.525
Nothing per mouth	92 (68.7%)	13 (65.0%)	59 (70.2%)	20 (66.7%)	0.871
Enteral feeding	83 (61.9%)	11 (55.0%)	55 (65.5%)	17 (56.7%)	0.547
Parenteral feeding	5 (3.7%)	1 (5.0%)	3 (3.6%)	1 (3.3%)	0.947
Medications:					
Gentamicin	1 (0.7%)	0 (0.0%)	1 (1.2%)	0 (0.0%)	0.741
Amikacin	8 (6.0%)	2 (10.0%)	6 (7.1%)	0 (0.0%)	0.261
Neomycin	1 (0.7%)	0 (0.0%)	1 (1.2%)	0 (0.0%)	0.741
Paromomycin	1 (0.7%)	0 (0.0%)	1 (1.2%)	0 (0.0%)	0.741
Esomeprazole	90 (67.2%)	13 (65.0%)	58 (69.0%)	19 (63.3%)	0.828
Furosemide	55 (41.0%)	11 (55.0%)	35 (41.7%)	9 (30.0%)	0.209
Thiazide	4 (3.0%)	1 (5.0%)	2 (2.4%)	1 (3.3%)	0.819
Amphotericin B	12 (9.0%)	4 (20.0%)	4 (4.8%)	4 (13.3%)	0.064
Tacrolimus	2 (1.5%)	1 (5.0%)	1 (1.2%)	0 (0.0%)	0.336
Electrolytes:					
Albumin, mean (SD), mmol/L	30 (6.8)	30.5 (7.5)	29.8 (6.7)	30.3 (6.7)	0.892
Corrected calcium, mean (SD), mmol/L	2.16 (0.21)	2.15 (0.15)	2.16 (0.20)	2.17 (0.28)	0.966
Sodium, mean (SD), mmol/L	141.1 (8.1)	140.4 (8.0)	140.3 (7.9)	143.8 (8.3)	0.107
Potassium, IQR, mmol/L	4.1 (3.8–4.6)	4.0 (3.6–4.5)	4.1 (3.8–4.6)	4.4 (3.8–4.9)	0.2061
Chloride, IQR, mmol/L	105 (100–109)	103 (100.5–108.5)	103 (100–108)	107.5 (105–115)	0.018
Phosphate, IQR, mmol/L	1.2 (0.93–1.64)	1.12 (0.86–1.32)	1.2 (0.94–1.62)	1.37 (1.04–2.44)	0.059
Hypomagnesemia Treatment (Mg replacement):					
Mg replacement	39 (29.1%)	11 (55.0%)	23 (27.4%)	5 (16.7%)	0.091
Total dose of IV Mg replacement; IQR, gram	0 (0–2)	1 (0–2)	0 (0–2)	0 (0–0)	0.034

QR: Interquartile Range; MIV: Mechanical Invasive Ventilation; NIV: Non-Invasive Ventilation; AKI: Acute Kidney Injury; ESRD: End-Stage Renal Disease; SD: Standard Deviation.

**Table 3:** Patient clinical outcomes based on ionized Mg concentrations on day of admission to ICU/HDU.

Characteristics n (%) Unless Specified Otherwise	Total 134 (100%)	≤0.46 mmol/L 9 (6.7%)	0.47–0.68 mmol/L 72 (53.7%)	≥0.69 mmol/L 53 (39.6%)	*p*-Value
Length of stay in hospital, IQR, (Days)	13 (5–32)	10 (4–11)	13 (5–31)	18 (7–41)	0.137
Length of stay in ICU/HDU, IQR, (days)	5 (2–13)	4 (2–6)	6 (2–13)	5 (2–14)	0.379
Time on ventilator days, IQR, hours	4 (2–7)	2 (1–5)	4 (2–7)	4 (2–7)	0.407
Seizure	21 (15.7%)	0 (0.0%)	12 (16.7%)	9 (17.0%)	0.408
Atrial fibrillation	32 (23.9%)	**4** (44.4%)	22 (30.6%)	6 (11.3%)	0.015
Ventricular fibrillation	2 (1.5%)	0 (0.0%)	2 (2.8%)	0 (0.0%)	0.417
Ventricular tachycardia	5 (3.7%)	1 (11.1%)	3 (4.2%)	1 (1.9%)	0.386
All type of arrhythmia	35 (26.1%)	5 (55.6%)	24 (33.3%)	6 (11.3%)	<0.01
90-day re-admission	18 (13.4%)	0 (0.0%)	9 (12.5%)	9 (17.0%)	0.363
In-hospital mortality	59 (44.0%)	4 (44.4%)	36 (50.0%)	19 (35.9%)	0.289
90-day mortality	61 (45.5%)	4 (44.4%)	37 (51.4%)	20 (37.7%)	0.317
ICU re-admission	15 (11.2%)	0 (0.0%)	12 (16.7%)	3 (5.7%)	0.275

IQR: Interquartile Range; ICU: Intensive Care Unit; HDU: High Dependency Unit; IQR: Interquartile Range.

**Table 4:** Patient clinical outcomes based on total Mg concentrations on day of admission (N = 134).

Characteristic n (%) Unless Specified Otherwise	Total 134 (100%)	≤0.69 mmol/L 20 (14.93%)	0.70–1.00 mmol/L 84 (62.69%)	≥1.01 mmol/L 30 (22.39%)	*p*-Value
Length of stay in hospital, IQR, (Days)	13 (5–32)	23.5 (14.5–66.5)	12 (5–24)	13 (4–41)	<0.01
Length of stay in ICU/HDU, IQR, (days)	5 (2–13)	8.5 (4.5–19)	4.5 (2–12)	5.5 (2–11)	0.073
Time on ventilator days, IQR, hours	4 (2–7)	6 (1−7)	3 (1.5−7)	4 (2–7)	0.278
Seizure	21 (15.7%)	6 (30%)	9 (10.7%)	6 (20%)	0.078
Atrial fibrillation	32 (23.9%)	5 (25.0%)	23 (27.4%)	4 (13.3%)	0.299
Ventricular fibrillation	2 (1.5%)	1 (5.0%)	1 (1.2%)	0 (0%)	0.336
Ventricular tachycardia	5 (3.7%)	1 (5%)	3 (3.6%)	1 (3.3%)	0.947
All type of arrhythmia	35 (26.1%)	5 (25%)	26 (31.0%)	4 (13.3%)	0.168
90-day re-admission	18 (13.4%)	3 (15%)	12 (14.3%)	3 (10%)	0.819
In-hospital mortality	59 (44.0%)	6 (30%)	39 (46.4%)	14 (46.7%)	0.391
90-day mortality	61 (45.5%)	6 (30%)	41 (48.8%)	14 (46.7%)	0.313
ICU re-admission	15 (11.2%)	4 (20%)	9 (10.7%)	2 (6.7%)	0.547

**Table 5:** Patient clinical outcomes based on fraction [(ionized Mg/total Mg) × 100] concentrations on day of admission to ICU/HDU.

Characteristic n (%) Unless Specified Otherwise	Total 134 (100%)	≤60%, 21 (15.67%)	>60%, 113 (84.33%)	*p*-Value
Length of stay in hospital, IQR, (Days)	13 (5–32)	6 (4–11)	16 (7–34)	<0.01
Length of stay in ICU/HDU, IQR, (days)	5 (2–13)	5 (2–7)	5 (2–13)	0.184
Time on ventilator days, IQR, hours	4 (2–7)	3 (2–7)	4 (2–7)	0.489
Seizure	21 (15.7%)	2 (9.5%)	19 (16.8%)	0.399
Atrial fibrillation	32 (23.9%)	5 (23.8%)	27 (23.9%)	0.993
Ventricular fibrillation	2 (1.5%)	1 (4.8%)	1 (0.9%)	0.178
Ventricular tachycardia	5 (3.7%)	1 (4.8%)	4 (3.5%)	0.786
All type of arrhythmia	35 (26.1%)	7 (33.3%)	28 (24.8%)	0.413
90-day re-admission	18 (13.4%)	3 (14.3%)	15 (13.3%)	0.901
In-hospital mortality	59 (44.0%)	13 (61.9%)	46 (40.7%)	0.072
90-day mortality	61 (45.5%)	13 (61.9%)	48 (42.5%)	0.101
ICU re-admission	15 (11.2%)	1 (4.8%)	14 (12.4%)	0.585

## Data Availability

The necessary data is provided in the Section 3, and more data can be obtained by contacting the corresponding author upon reasonable request.
